# Pushing the boundaries: Uterine transplantation and the limits of reproductive autonomy

**DOI:** 10.1111/bioe.12531

**Published:** 2018-10-15

**Authors:** Laura O’Donovan

**Keywords:** absolute uterine factor infertility, assisted reproduction, reproductive autonomy, reproductive technologies, uterine transplantation

## Abstract

Over the course of recent years, various scientific advances in the realm of reproduction have changed the reproductive landscape, enhancing women’s procreative rights and the choices available to them. Uterus transplants (UTx) are the latest of such medical innovations aimed at restoring fertility in women suffering from absolute uterine factor infertility, providing them with the possibility not only of conceiving a genetically related child but also of gestating their own pregnancies. This paper critically examines the primacy of reproductive liberty in the context of uterus transplantation. It questions whether and to what extent we should respect the reproductive autonomy of a woman who chooses UTx, given the significant risks that attach to the procedure and existing concerns that UTx may perpetuate potentially troubling gendered norms surrounding pregnancy and the role of women’s bodies in reproduction, which may place undue reproductive pressures on women.

## 1 INTRODUCTION

Over the course of recent years various scientific advances in the realm of reproduction have changed the reproductive landscape, enhancing women’s procreative rights and the choices available to them. Uterus transplants (hereafter UTx) are the latest of such medical innovations aimed at restoring fertility in women suffering from absolute uterine factor infertility (AUFI), providing them not only with the possibility of conceiving a genetically related child, but also of gestating their own pregnancies. It is estimated that AUFI affects approximately one in 500 reproductive‐age women worldwide,^1^ for many of whom infertility has devastating consequences. Currently, women with AUFI are ‘considered as being “unconditionally infertile”’.^2^ Traditional methods of assisted reproduction are unable to treat infertility of this nature, and the only options available to such women seeking to found families are adoption or surrogacy. UTx in this sense aims to provide women suffering from AUFI with a third option in the management of their infertility. Proponents of the procedure emphasize this unique choice‐enhancing potential, whilst detractors focus on the medical and psychological risks it presents. Fundamentally, the issue of the permissibility of UTx comes back to one of the limits of autonomy.

This paper critically examines the primacy of reproductive autonomy in the context of uterine transplantation. It questions whether and to what extent we should respect the reproductive autonomy of a woman who chooses UTx. It explores two of the main reasons for allowing UTx: in Section 2, that UTx offers something of positive experiential value over and above alternative methods of family creation, and in Section 3, that offering the procedure to women who choose it respects their reproductive autonomy, which (with some caveats [Section 4]) is something we ought to do. It then turns to examine two of the main arguments against allowing UTx: in Section 5, that the procedure presents various harms of a physical and psychological nature to the live donor, recipient and children created by it, and in Section 6, that there are concerns that UTx may perpetuate potentially troubling gendered norms surrounding pregnancy and the role of women’s bodies in reproduction, which may place undue reproductive pressures on women.^3^ It comes to the conclusion that on current available evidence, UTx arguably meets the harm/benefit threshold such that in order to respect the reproductive autonomy of the woman who chooses it, the procedure ought to be permitted.

## 2 EXPERIENTIAL VALUE OF UTERINE TRANSPLANTATION

In order to fully appreciate the nature of the issues at stake for women who may seek UTx, it is important to consider the value that the procedure may hold in both an experiential and a symbolic sense. AUFI is defined as the ‘absence of the uterus, or the presence of a non‐functional uterus’.^4^ The condition is thought to affect more than 12,000 women of childbearing age in the U.K. alone, with causes attributed to various conditions from the genetic disorder Mayer–Rokitansky–Küster–Hauser syndrome to cervical cancer.^5^ For women with AUFI who wish to experience motherhood, their only recourse is either adoption or surrogacy (where legally permitted). For some women, the presence of a genetic and/or gestational link between mother and child may be of little importance, and adoption offers a satisfactory route to motherhood. For others, a genetic link may be of more significance, and in this case gestational surrogacy (where the implanted embryo is created using the intended mother’s and father’s gametes) is currently the only way to achieve this.

It is self‐evident that the experience offered by uterus transplant is unique, aiming to parallel as closely as possible the reproductive experience of the naturally fertile woman, in the sense that a woman undergoing UTx will gestate and carry to term a pregnancy, looking no different from her fertile pregnant counterpart to the outside world. This is something that surrogacy and adoption simply cannot provide. For those who view the pregnancy experience as providing something of value, this type of transplant captures what some women with AUFI may perceive to be the ‘authentic’ motherhood experience. Society undoubtedly places a high value on the pregnancy experience, though this is not only relevant to the way we perceive motherhood: UTx has also been described as offering ‘a way for these women to *re‐discover their own femininity* through the restoration of fertility’.^6^

Indeed, societal and family pressures and personal expectations regarding procreation may place both a physical and a psychological burden on the infertile woman,^7^ leading to feelings of hopelessness and depression.^8^ Whilst gestational surrogacy (where permitted) and adoption may provide solutions for some women seeking to have children, for others these options may be unacceptable, or at least constitute less appealing alternatives. This is particularly so where various concerns of a legal,^9^ ethical and financial nature are present and heightened by a preference for a biological, physical link between parent and child, which is embodied in the gestational experience. It is for this reason that even a woman who is able to have a genetic child through surrogacy may still prefer to undergo UTx to enable her to fulfil the desire not just for ‘[her] own child, but for [her] own pregnancy’.^10^ In this sense, surrogacy and adoption may be viewed as imperfect substitutes for UTx owing to the lack of the experiential dimension, which may be demonstrably valuable to women. This might give us reason to suggest that increasing the number of options available to women by offering transplant may serve to enhance their wellbeing.

Increased choice alone, however, is of purely formal benefit if an individual has neither the capacity nor the opportunity to exercise such choice.^11^ Having set out why UTx may be something that appeals to some women with AUFI, we must now turn to examine the concept of reproductive autonomy in both a moral and a legal sense, and the related notion of procreative liberty. Only then may we begin critically to question the parameters of reproductive decision‐making in the transplantation context.

## 3 AN ACCOUNT OF REPRODUCTIVE AUTONOMY

The idea that autonomy can constitute an organizing principle in the field of reproduction is not new.^12^ In its simplest form, autonomy can be understood as the idea that individuals ought to be able to make decisions with respect to their life projects according to their own beliefs and wishes,^13^ insofar as others are not harmed by the exercise of their decision‐making. As Jackson points out, however, it would ‘be a mistake to imagine that the concept of autonomy within the liberal tradition had a single, unitary meaning’.^14^ Instead, she argues:

[A] variety of different ideas concerning moral independence, self‐government, freedom from external constraints, tolerance, pluralism and liberty have all crystallised around the notion that an individual’s life may be enriched by her capacity to direct the course of her life according to her own values.^15^

Arguably, given the intimate nature of reproductive decision‐making, involving decisions as to when, with whom and how one might reproduce, and the significance that these decisions may have on an individual’s sense of self, dignity and wellbeing, there is scope to suggest that society ought to strive to respect and uphold reproductive autonomy as far as it is possible to do so. What might restrict or encumber the exercise of autonomy in the traditional liberal sense is an account of harm, most notably developed by Mill, whose ‘harm principle’ tells us that a person is sovereign over his own life up to the point where others’ welfare may be threatened.^16^ When it comes to reproductive decisions, adherence to this version of the liberal tradition would say that those decisions are the sole domain of the individual—that the state has no role to play except in those circumstances where another may be harmed. This other may be the prospective child, and ‘harm’ may potentially be interpreted quite widely. Although it is beyond the scope of this paper to provide a thorough critique of the harm principle, it suffices to state that a version of it (that identifies ethical concerns, tangible physical harms, e.g. risks of physical injury, and harms of social injustice, balanced against the identifiable benefits of a purported treatment) seems to guide the approach to reproductive treatment taken by English law. A recent example of this can be found in the case of mitochondrial replacement therapy, where lengthy deliberation of the harm/benefit ratio took place, involving a government public consultation exercise and detailed reports by science and ethics committees,^17^ leading to debates in both houses of Parliament. This culminated in Parliament’s acceptance of the merits of treatment and with legal provision being made for the regulated use of mitochondrial donation,^18^ notwithstanding the concern raised that the safety of mitochondrial donation is ‘uncertain and in absolute terms will remain so until several generations of people have been born from the procedure’.^19^

A further criticism levelled against the standard account of autonomy suggests that viewing autonomy primarily as a commitment to one’s project of self‐making is excessively individualistic and ignores our interrelatedness.^20^ Certain commentators have argued that dichotomizing ideas about autonomy and interdependence with others promotes an overly simplistic, narrow view of what it means to be autonomous. 21 Autonomy, on these accounts, ought not to be understood as a one‐dimensional promotion of the self as an insular being, divorced from the multifaceted network of relationships and socio‐cultural influences that make up an individual’s life—the thing that distinguishes them from you or me.^22^ Instead, subscribing to a richer account of autonomy, which acknowledges the ‘combination of individuality and “enmeshedness”, integrity and integration that constitutes the human being’,^23^ seems preferable in the sense that it more accurately reflects our lived experiences of autonomy. I start then from a position on reproductive autonomy that acknowledges the importance of being able to direct one’s life plan according to one’s own values and beliefs, granted that they may have been shaped by, or arisen as a result of, our multiple connections with others. It is arguable therefore, that if a woman has a strong desire vis‐à‐vis her reproduction, for example a desire to gestate, and this holds some value for her, then it will be in her interests to do so in some morally meaningful way. Whilst it has been argued that reproductive decisions are decisions of a particularly important and intimate nature, given the fact that they will undoubtedly have a significant effect on the way in which individuals direct the course of their lives,^24^ (and indeed there might be debate about whether this is capable of recognition as a critical or experiential interest in the Dworkinian sense^25^), what counts for the present purpose is not necessarily the reproductive nature of the decision *per se* so much as the interest we all have in leading a life in accordance with our own values.

Related to the concept of autonomy is the notion of what Robertson terms procreative liberty.^26^ Liberty in this sense refers to the sphere within which an individual may make choices—their capacity and ability to exercise reproductive decision‐making. In other words, liberty is not synonymous with autonomy, but it does encompass the notion of autonomy, in that an individual is able to make reproductive choices only when at liberty to do so. Robertson defines procreative liberty at its most general level as ‘the freedom to either have children, or to avoid having them’.^27^ Establishing a theoretical framework for his understanding of the concept, he states:

The moral right to reproduce is respected because of the centrality of reproduction to personal identity, meaning and dignity. This importance makes the liberty to procreate an important moral right, both for an ethic of individual autonomy and for the ethics of community or family that view the purpose of marriage and sexual union as the reproduction and rearing of offspring. Because of this importance the right to reproduce is widely recognised as a prima facie moral right that cannot be limited except for very good reason.^28^

‘Good reason’ here relates to the general liberal concept of harm described above. Robertson suggests that reproductive choices may be limited if the exercise of procreative liberty creates ‘substantial harm to the tangible interests of others’, where the burden of demonstrating this ‘lies with those who would limit procreative choice’.^29^ Where there are both identifiable benefits and harms established for a particular treatment, a balancing exercise is required. As we shall see, with UTx, this necessarily involves weighing the potential harm caused to the live donor, transplant recipient and fetus in gestating a pregnancy in a transplanted womb against the harm of denying the interests of the woman who wishes to found her family in this way. Autonomy or liberty as moral rights are only formal unless they are protected (by ensuring that choices are respected) and supported (by ensuring that choices are provided for in some way) in law and policy. In English law, for all the reasons outlined previously relating to the idea that a good society is one that attempts to facilitate an individual’s life choices, autonomy is arguably protected if we consider that an individual may consent or refuse consent to treatment. In principle, the law will not interfere with treatment decisions (where the patient has capacity).^30^ Whether autonomy is supported in the sense that choices are provided for, however, warrants further consideration.

Reproductive liberty such as Robertson envisages is essentially a negative construction—rights against the interference from other individuals (or the state) in the exercise of one’s reproductive choices unless this causes harm. Intervention remains a matter of resource allocation and broader social policy.^31^ Writing in defence of reproductive autonomy, Jackson draws on the work of Raz: ‘to be autonomous a person must not only be given a choice but he must be given an adequate range of choices’.^32^ Applying this to the context of reproductive services, Jackson argues that:

[P]roper respect for reproductive autonomy cannot [therefore] be limited to removing external constraints from an individual’s capacity to follow preferences that are *already* fully formed and clearly articulated. Instead there may be times when positive provision of resources and services may be necessary in order to assist people both to work out their own priorities and to realise them.^33^

Of course, offering treatment in a free healthcare system is one thing; controlling who has access to ensure the fair distribution of resources is another and often leads to demand far outstripping the available supply—the patchy provision of IVF services in the U.K. being a clear case in point here.^34^ The English Court of Appeal in *Burke *held that ‘autonomy and the right of self‐determination do not entitle the patient to insist on receiving a particular medical treatment regardless of the nature of the treatment’.^35^ As we might suspect then, in practical terms, given the fact that the National Health Service (NHS) is a publicly funded healthcare system, for reasons of distributive justice and fair rationing policy, the state simply cannot facilitate an unfettered right of access to assisted reproductive technology (ART),^36^ and so any formal reproductive rights (underpinned by notions of reproductive liberty and autonomy) in English law should be understood as negative rights to non‐interference.^37^

Having analysed the concept of reproductive autonomy in both a moral and a legal sense, we now have a point from which we can begin to evaluate the purported value of UTx (as mentioned above) versus the known risks of complication and potential harm that may be caused by the procedure, in order to be able to come to an informed conclusion on the primacy and limits of reproductive autonomy in the UTx context.

## 4 THE LIMITS OF REPRODUCTIVE AUTONOMY

As noted earlier, following a Millian account of liberty, an individual is generally regarded as sovereign over his own life, unless the benefits of making autonomous choices in accordance with one’s own project of self‐making are outweighed by the risk of harm to others. Inasmuch as that society has a moral reason to respect the autonomous decisions of individuals, it also has a duty to prevent harm to others, and it is this duty that grounds society’s interest in the direction of assisted reproduction (AR). If the risks of harm posed by UTx are deemed to be of such significance that they outweigh the benefits, then UTx will be unacceptable and should not enter routine practice. Correspondingly, if English law is broadly Millian in its approach, we would expect it to be wary of UTx. Having said that, if there is no reason to think that UTx would be especially harmful to others, the presumption would be that it ought to be allowed.

The potential harms that UTx poses to the live donor, recipient and developing fetus can be categorized as physical and psychological. Other concerns relate to the possibility of social pressures conditioning a woman’s preferences and desires to the extent that this may actually serve to undermine reproductive autonomy. (Considerations relevant to UTx such as those relating to donation, resourcing issues in a publicly funded healthcare system and the deeper debate about the proper aim of medical treatment fall outside the scope of the present analysis.) The risk of harm to each relevant party will now be examined in turn, before concerns regarding the perpetuation of potentially troubling gendered norms are explored.

## 5 THE POTENTIAL HARMS POSED BY UTx

### 5.1 Harm to live donor

In June 2018, Mr Richard Smith, clinical lead at Womb Transplant U.K., announced plans to extend the U.K. UTx trial to include two live donor operations.^38^ Initially, NHS Blood and Transplant approved the clinical trial in 2015 on the basis that organs would be procured from deceased donors, obviating many of the concerns that exist with live donation, including mainly, but not limited to, risks of physical or psychological harm to the health of the donor and issues regarding the possibility of coerced consent. Given the recent trial update to include live donors, it is imperative that risks here are examined^39^ and included in the harm/benefit assessment of the acceptability of the procedure.

#### 5.1.1 Risk of harm and defective consent

The risk of physical and psychological harm to the live donor is evident. Donation requires that she undergo hysterectomy, a major abdominal surgery that is not clinically necessary. Whilst this poses no risk in deceased donation, as Williams points out, ‘retrieval in live donation *necessitates *physical harm to the donor and includes small but not insignificant risks of long‐term morbidity and mortality thought similar to, or only slightly higher than that of a total abdominal hysterectomy’.^40^ In the Swedish trial, it was reported that the mean operative time for donor surgery was 11.6 hours, compared with 4.7 hours for the recipient surgery.^41^ Surgical risk remains one of the major concerns with the procedure, particularly for the donor, given the highly invasive nature of the operation to retrieve the uterine veins and the length of the operation, meaning that general anaesthetic is required.^42^ It is hoped that the use of robotic‐assisted surgery will significantly reduce donor surgery time. In 2017, a team from Xi’an, China reported that graft procurement with robotic assistance significantly reduced surgery time to 6 hours.^43^ Further physical health risks include the risk of post‐operative complications including infection, thrombosis, fistula^44^ and uretic injury.^45^ Psychological problems unique to UTx comprise issues relating to gender identity, feminine self‐image and sexual dysfunction, all of which may result in depressive symptoms.^46^ Furthermore, generalized psychological issues that present a risk factor in live organ donation include anxiety, depression and stress, which may be complicated further by perioperative pain.^47^

However, whilst the uterus retrieval procedure clearly poses tangible risks to the health of the donor, it should be noted that other live donation procedures pose similar risks. Consider the history of liver and kidney transplantation from live donors. Perioperative complications such as bleeding and infection are also present, and in the case of liver transplants, surgical time has halved over the years from a mean initial time of 12 hours to five or six hours today.^48^ It would be unduly paternalistic for the state to intervene in an individual’s decision to donate on the basis of the surgical risks identified, where similar risks do not serve as justification to prevent donors of livers or kidneys from donating. Where risks are manageable, and the procedure has some corresponding benefit for the recipient, it ought to be for the individual donor herself to weigh up the benefits and risks of the procedure. Whilst benefit in the case of UTx may be harder to identify, given that the procedure is non‐life‐saving in purpose, it exists in the form of a positive psychological experience—the satisfaction of having enabled the recipient to attempt to carry her own pregnancy.

Organ donation in the U.K. operates on the basis of an altruistic model. Under English law, the decision to donate must be voluntary, informed, and the patient must have capacity to consent to the procedure following the Mental Capacity Act 2005.^49^ The risk of coerced or induced consent is not unique to uterine transplantation. The NHS has robust counselling and assessment procedures in place for other forms of live donation to ensure that consent is valid and that no reward for donation has been sought,^50^ and this would undoubtedly extend to UTx. It is true that the nature of the transplant differs somewhat from that for kidneys and livers, in that the donor has to be sure that they have completed their own family and no longer requires the organ. In this sense, whilst an enhanced counselling and consent process may be required to ensure that donors fully comprehend the nature and consequences of their decision, there is no reason to think that unique concerns here provide sufficient reason to prohibit the procedure. Arguably, the benefits of respecting the reproductive autonomy of both the donor, in deciding that she no longer wishes to have any more children and is prepared to lose her biological capacity to do so, and the recipient, in wishing to constitute her family in a way that is meaningful to her, sufficiently outweigh the potential negative consequences.

### 5.2 Harm to recipient and fetus

#### 5.2.1 Risk of physical harm

Compared with existing reproductive technologies, UTx necessarily involves a significantly larger, more invasive degree of medical intervention, comprising oocyte retrieval, uterus transplant and immunosuppressive medication, single embryo transfer, birth by caesarean section, and eventual hysterectomy given the ephemeral nature of the transplant. (On this last point, it is important to note that most models of UTx assume that the uterus would be removed after a certain period—see Brännström.)^51^ Daar and Klipstein summarize the physical risks to woman and developing fetus as follows:

In order to achieve pregnancy via uterine transplantation, the uterine vessels need to be reconnected to those of the recipient. This delicate surgical technique poses risks of blood clots and compromised blood supply, which can negatively affect fetal growth. In addition, since the organ is transplanted, the recipient of the uterus requires varying levels of medication to prevent rejection of the organ. These medications not only increase her risk of malignancy over time, but cross the placenta. While most of the immunosuppressive medications have not been shown to increase the rate of birth defects (and those that do can be avoided during pregnancy), several are associated with low birth weight and preterm delivery.^52^

They rely on the above health risks to justify their argument that, applying the principle of procreative beneficence, selecting the best child possible as one gestated in a transplanted womb seems inferior to one gestated in the native uterus of a surrogate.^53^ Furthermore, they conclude by urging a ‘refocusing [of] the debate over womb transplants from one steeped in reproductive liberty to one that considers the well‐being of a future child destined for birth with the aid of modern technologies’.^54^ Here, I argue that interest in respecting reproductive autonomy and valid concern regarding the risk of harm to both woman and developing fetus are not mutually exclusive. Refocusing the debate to centre on the latter concern risks understating the importance of the former. Instead, detailed analysis of both is required. Whilst we are right to be concerned about evidence of risk to the health of a developing fetus in a UTx pregnancy, it should be noted that the main risks identified are also present in other pregnancies to varying degrees. Consider, for example, the case of pregnancy in recipients of solid organ transplants. The risks of immunosuppression therapy in patients undergoing UTx are similar to those in recipients of other solid organs,^55^ and we do not consider this as a means by which to prevent solid organ transplant recipients from seeking AR services, or reproducing naturally. Thus, it is unclear how one might justifiably distinguish UTx from pregnancy in other cases of solid organ transplant at this stage. Whilst it is acknowledged that generally long‐term observational data sets are limited, ‘registry records and case reports [up to 2006] have not been able to find unifying patterns of [structural] malformations in children of recipients of solid organs’.^56^

This, however, is not to ignore data relating to the incidence of premature birth and low birth weight in transplant recipients, which warrants careful consideration.^57^ Indeed, both prematurity and low birth weight are known to increase the risk of infant morbidity and mortality.^58^ In pregnancy outcomes recorded on the U.K. Transplant Pregnancy Registry, of 149 live births between 1994 and 2001, 51% were premature and 54% of babies born were of low birth weight.^59^ Preterm delivery and low birth weight occurred in both the reported live births by UTx in Sweden.^60^ However, follow‐up studies on the two children reported that for the first child ‘the first postnatal week was uneventful and the baby was in good condition, requiring only phototherapy and room air … [and] was discharged in good health from the neonatal unit 16 days after birth’^61^, whilst the second baby ‘developed fully normally, with weights (kg) of 4.0, 6.2, 7.4, and 9.3 at ages 2, 4, 6, and 12 months, respectively’.^62^ To date there have been a total of eight children born^63^ as a result of the Swedish trial; however, before any conclusions can be drawn on the safety and efficacy of UTx in terms of fetal health/child development, further data must be obtained. Currently, there is no evidence to suggest that pregnancy outcomes in UTx differ from those in other transplant recipients.

The risk of harm to the health of the woman as a result of immunosuppression therapy is mitigated somewhat owing to the temporary nature of uterine transplantation, with removal recommended after one or two healthy live births.^64^ Other major risks are associated with surgical complications and anaesthesia. For example, following the hysterectomy procedure performed on the second transplant recipient to give birth in the Swedish trial, Brännström et al. reported finding ‘few adhesions around the uterine fundus, but extensive paracervical adhesions were present … [furthermore], one month after hysterectomy, the patient developed signs of infected vaginal vault hematoma … [requiring] transvaginal drainage and oral antibiotics’.^65^ It should be noted, however, that the same patient was reported to be in good health 12 months after childbirth.^66^ Whilst these data present concrete evidence of the health risks posed by UTx, in the exercise of reproductive autonomy, the question whether a woman who would choose this treatment ought to be able to navigate risks to her health alone is raised. Indeed, more generally when it comes to healthcare decisions, as previously stated, English law will not (usually) interfere with a patient’s treatment decisions.^67^ Of course, however, the issue is not as simple in the case of UTx, given the potential risks that the treatment poses to the health of the developing fetus and future child. Although the fetus has no legal personality in law,^68^ it may hold some degree of moral significance,^69^ and it is for this reason that wider society has an interest in determining the acceptability of the procedure.

#### 5.2.2 Risk of psychological harm

Regarding the risk of psychological harm to the future child owing to the atypical nature of gestation and birth, at this stage and absent evidence to suggest otherwise, similarities may be drawn from the psychosocial experience of children born through surrogacy. Longitudinal studies of surrogate‐born children (by IVF or artificial insemination) demonstrate that ‘there were no significant differences in children’s total SDQ [strengths and difficulties questionnaire] scores between family types for either mothers’ or teachers’ ratings’.^70^ On the possibility of bias in maternal reporting of child behaviour, the authors note that the teacher questionnaire ‘provided an independent rating of the presence of emotional or behavioral problems in the children that confirmed the mothers’ reports’.^71^ Any such concerns may be more acute in cases of possible future transgender UTx and male UTx, but current research involves transplant only in female patients. Again, at this stage in the research, it is not possible to determine whether being born by UTx may cause psychological harm, similar to, or above, that which may be experienced by children born through surrogacy and/or IVF, and whether any such harm is significant to the extent that the procedure should not be allowed. This is something that can only be assessed as further research and longitudinal studies are carried out. It is not, however, sufficient reason to prohibit the availability of UTx at the clinical research stage where the data required may be readily obtained.

Of more significance is the possibility of psychological harm to the woman seeking UTx. It is well documented that there is a greater risk of psychological disorder in transplant candidates and recipients over and above the general population,^72^ and this, combined with the possibility that some form of psychological condition may already be indicated in a woman with AUFI as a result of a diagnosis of infertility,^73^ means that it is imperative that stringent vetting and consent procedures are in place to ensure that a woman’s capacity to exercise such choice is not compromised. Again, there is little information available from which we may confidently draw conclusions, owing to the relative newness of the procedure, although researchers including the Swedish team aim to perform ‘continuing long‐term psychological follow‐up of the recipients and their partners and donors’.^74^ Limited data from Sweden show that in a one‐year follow‐up study post‐transplant, ‘the participants of the trial at baseline showed that they were all individuals with comparable or better psychological well‐being than norm populations’^75^, and although recipients with ‘ongoing graft showed significant stress 3 months after UTx, and [2 with graft failure] reported lower physical functioning and ongoing bodily pain, [this] … returned to baseline 6 months past surgery.’^76^

In a recent study^77^ undertaken by the British UTx research team investigating the psychological issues associated with AUFI and patients’ attitudes towards transplant, it was found that after being provided with information explaining the risks and benefits of all three fertility options available (including UTx), 92.5% of women interviewed stated that they ‘would undergo UTx (or would have in the past) as a first‐choice treatment (ahead of surrogacy and adoption) for AUFI, provided it was a safe and accepted procedure in the medical literature’.^78^ It should be noted, however, that when the possibility of graft failure and potential risk to the fetus posed by the immunosuppressive regime were introduced, the proportion of women who stated they would opt for UTx fell to 87.5%.^79^ Whilst gestational motherhood is the ultimate goal of UTx^80^, clearly not all women are willing to embrace the risks of the procedure in order to experience pregnancy. However, data here should be interpreted with caution given the possibility of bias considering the limited sample size^81^ of women interviewed, and the pool from which women were contacted for interview—namely women who had contacted Womb Transplant U.K. and expressed an interest in UTx.

It is clear from the data collated that UTx offers something that a large proportion of women with AUFI may want. However, concerns exist regarding the possibility that the intense desire to procreate may lead to women failing to appreciate fully the nature of the risks involved in the procedure and undergoing an operation that they may not have otherwise had. Indeed, the results of the aforementioned study led by the Womb Transplant U.K. team, concluded that:

[R]ather worryingly, despite being warned about the risks of UTx, patients are potentially distancing themselves from them in order to achieve their goal of fertility. They are so focused on improving their QoL that their views on UTx and whether it may be right for them become 1‐dimensional to the extent where they would undergo UTx, ignoring the potential harm to themselves and the unborn fetus.^82^

It is right to be concerned here, and this may be a place where we would at least want to consider limiting reproductive liberty. Consider, for example, the case of multiple embryo transfer (MET) and the health risks this poses to both mother and developing fetus.^83^ A woman may desire a twin or triplet pregnancy; we know, however, that multiple births are associated with a higher rate of risk of complications in the form of prematurity, low birth weight, congenital anomaly, high blood pressure and preeclampsia etc., and so we may have a reason to actively counsel against pursuing such a pregnancy, especially where the patient is unable to fully appreciate the nature of the risks involved. Indeed, current HFEA (Human Fertilisation and Embryology Authority) policy^84^ does not recommend MET, and consequently many clinics routinely only offer double embryo transfer in exceptional circumstances. This scenario may represent an example of the limits of reproductive autonomy; however, it is arguably distinguishable from the case of UTx in that whilst the desire for twins, triplets or quadruplets may not be satisfied in refusing MET, the correlating desire to gestate and parent *is* satisfied. By contrast, in denying the autonomous choice of a woman in the UTx context, and assuming that adoption and surrogacy are unavailable, she cannot even attempt to satisfy her desire to be a genetic and gestational parent at all. Furthermore, we are currently considering transplant in the very limited clinical trial context, where potential candidates receive extensive counselling to ensure that they understand the purported benefits and risks of treatment before providing consent (although it is acknowledged that this is something that will require further empirical research). IVF is a routine medical treatment offered both nationally and abroad, and thus if MET was respected the potential harm caused would perhaps be significantly higher in that there are many more patients who may request it, especially if it is thought that this may increase the chances of becoming pregnancy.^85^

Of course, not everyone has the opportunity to satisfy his or her desires. However, where the procedure exists and trials are taking place, it seems arguable that there is at least a prima facie case to suggest that we ought to respect the reproductive preferences of a woman who chooses UTx. Pregnancy in a native uterus is not without risk,^86^ and whilst the risk of possible complications may be increased in the case of UTx, current available evidence does not show that the risks of harm outweigh the benefits to be derived from the procedure, at least at the clinical trial stage. It is important for the women whom this procedure may assist that we do not allow doubt as to the efficacy and safety of treatment based on what is *not* known to impede empirical research. If, however, following further research, such risks are demonstrably more significant in UTx than in other options for family‐building open to women with AUFI (notably surrogacy), then UTx may not meet the requisite safety standards, namely harm threshold criteria, to enter routine clinical practice.

## 6 SOCIAL PRESSURE

Lotz writes:

[H]uman reproduction is a profoundly social phenomenon, deeply embedded in complex social norms and aspirations … [and] as such, reproductive technologies like UTx must be examined fully in light of their wider social impacts, as embodying and communicating significant values, and as occurring within a dynamic and reciprocal communicative relationship between state, society and individual.^87^

The tension between assisted reproduction and the social context within which treatment is offered has long been the topic of debate. There are those, who, like Firestone, would argue that ARTs have strengthened women’s autonomy in reproductive matters, allowing them to make a free and informed choice about if, when, and how to reproduce, while simultaneously providing a means of challenging the enduring primacy of the nuclear family.^88^ However, opposing this view to different degrees are those who suggest that ART may serve to compromise women’s autonomy and self‐determination in reproduction owing to the social pressure implicit in reproductive decisions, and the degree of external control that third parties continue to have over women’s reproductive choices through treatment provision.^89^ Following on from consideration of the medically risky nature of UTx, we may also be concerned whether, and to what extent, ‘the social factors and conditions including attitudes, desires, values and biases’^90^ that ‘the alleged benefits of UTx are inextricably bound up with’^91^ may serve to undermine the exercise of reproductive autonomy.

The idea that social norms and perceptions of the family may influence a woman’s reproductive decision‐making is not new. Concerns here relate to the pressure of the ‘motherhood mandate’^92^—women may feel compelled to turn to assisted reproduction in order to satisfy the genetic and gestational motherhood imperative when they may not otherwise have done so.^93^ Whilst no empirical research has yet been published identifying the motivating factors behind the decisions of the women signed up to the UTx trial in the U.K., the British transplant team has published a guide ‘patient pathway’ protocol,^94^ including questionnaire forms to be filled out by participants in the trial, with a section exploring patient motivation in opting for surgery.^95^

When analysing the extent to which choice in this context may be shaped by the social pressure to reproduce and experience pregnancy and childbirth, it is important to remember that UTx is not unique in this sense (although concerns here may be heightened). The decision to undergo IVF treatment encompasses many of the same issues, and, presumably, at least some of the same motivations also underpin this choice, and yet IVF is now an accepted common practice, with more than 240,724 babies born in the U.K. after IVF treatment between 1991 and 2013.^96^

Jackson neatly summarizes the interplay between reproductive choices and external influences when she states that, ‘individuals cannot exist in … a social and cultural vacuum … Reproductive decisions, in particular, will obviously be informed by the rich network of relationships and cultural expectations within which each individual is situated’.^97^ While this, of course, becomes a problem should women feel obliged to choose a certain option in order to fulfil certain societal and cultural expectations, we have no more reason to suspect that this is the reason women may choose UTx than the understandable desire to experience pregnancy. It is possible that to a woman with AUFI, UTx presents the better of two similarly ‘risky’ options when compared with surrogacy, the difference being that, as opposed to externalizing the risk of gestation in surrogacy and the legal complexities involved therewith, in UTx a woman chooses to internalize and assume that risk herself.

Generally, we do not seek to influence or curtail an individual’s choice in natural reproduction, nor do we question the authenticity of the same, citing concerns regarding social pressures of procreation, and thus in this sense we have no more reason to do so with regard to UTx as a treatment option. Thus, despite worries that may exist involving the perpetuation of gendered norms and cultural expectations, where a woman is provided with an additional (albeit risky) option to treat her infertility, the mere presence alone of such social and cultural norms does not necessarily preclude at least a prima facie claim that we should respect the reproductive autonomy of the woman choosing the procedure.

## 7 CONCLUSION

This paper set out to examine the primacy of reproductive autonomy and the question of whether we should respect the choice of a woman who desires UTx, thinking that she will achieve a greater sense of wellbeing while the opposite may be true, taking into consideration the risks of harm to the live donor, recipient and developing fetus. The nature and importance of autonomy was explored both morally and in law in order to unpick the backdrop of interests against which this new reproductive technology is set. As I indicated earlier in this paper, UTx is an experientially unique form of AR, which aims to treat a subset of female‐factor infertility previously deemed untreatable. It has increased the reproductive options available to women with AUFI who previously had recourse only to surrogacy or adoption to create their families. Current evidence does not suggest that the risks of harm posed by the procedure outweigh the potential benefits to be derived from respecting the choice to undergo donation and transplant—namely, the inherent value in respecting the autonomous decision of an individual to live a life in accordance with their wishes. At present, any conclusions presented here must be restricted to the clinical trial context, as further data collection is required in order to decide on the moral permissibility of treatment in routine clinical practice (similar to the process by which mitochondrial replacement therapy was evaluated here in the U.K.). This is not something that women should navigate alone, owing to the nature of the risks involved and the interest that society has in the direction of human reproduction. Reproductive decisions concern some of the most intimate spheres of our lives, and whilst society has an obligation to prevent harm to others, for as long as UTx meets the harm threshold and the possible risks of treatment do not outweigh the benefits, we ought to respect the reproductive autonomy of a woman who wishes to undergo the procedure.

## CONFLICT OF INTEREST

The author declares no conflict of interest.

## Notes

1 Järvholm, S., Johannesson, L., Clarke, A., & Brännström, M. (2015). Uterus transplantation trial: Psychological evaluation of recipients and partners during the post‐transplantation year. *Fertility and Sterility,* *104*(4), 1010–1015.

2 Saso, S., Clarke, A., Bracewell‐Milnes, T., Saso, A., Al‐Memar, M., Thum, M. Y., Yazbek, J., … Smith, J. R. (2016). Psychological issues associated with absolute uterine factor infertility and attitudes of patients toward uterine transplantation. *Progress in Transplantation, 26*(1), 28–39, p. 29.

3 See Petropanagos, A. (2016). Pronatalism, geneticism and ART. *International Journal of Feminist Approaches to Bioethics*, *10*(1), 119–147, where the author suggests that ‘social bias that favors childbearing, and geneticism, a social bias that favors genetic relatedness … may influence reproductive choices and unduly pressure some women into choosing expensive and medically risky ART’, at p. 120.

4 Brännström, M., Diaz‐Garcia, C., Johannesson, L., Dahm‐Kähler, P., & Bokström, H. (2015). Livebirth after uterus transplantation. *The Lancet*, *385*(9968), 607–616.

5 Ibid.

6 Saso, S., Bracewell‐Milnes, T., Ismail, L., Hamed, A. H., Thum, M. Y., Ghaem‐Maghami, S., & Del Priore G, Smith, J.R., (2014). Psychological assessment tool for patients diagnosed with absolute uterine factor infertility and planning to undergo uterine transplantation. *Journal of Obstetrics and Gynaecology, 34*(6), 504–507, p. 506 (emphasis added).

7 Monga, M. (2004). Impact of infertility on quality of life, marital adjustment and sexual function. *Urology*, *63*(1), 126–130.

8 Cousineau, T. M., & Domar, A. D. (2007). Psychological impact of infertility. *Best Practice & Research Clinical Obstetrics and Gynecology, 21*(2), 293–208.

9 In the U.K., the adoption process is a cumbersome one and various issues exist regarding the provision of legal parenthood in surrogacy arrangements through the grant of a parental order under s54 of the Human Fertilisation and Embryology Act 2008 in the U.K. context.

10 Catsanos, R., Rogers, W., & Lotz, M. (2013). The ethics of uterus transplantation. *Bioethics, 27*(2), 65–73, p. 65.

11 On this point, see Raz, J. (1986). *The morality of freedom*. Oxford: Oxford University Press; and Jackson, E. (2001). *Regulating reproduction: Law, technology and autonomy*. Oxford: Hart Publishing.

12 On this point, see Jackson, E. (2001). *Regulating reproduction: Law, technology and autonomy*. Oxford: Hart Publishing.

13 Note that the terms wish and desire are used interchangeably throughout this paper.

14 Jackson, *op. cit*. note 12, p. 2.

15 Ibid.

16 Mill, J. S. (1859). *On liberty*. Retrieved from https://ebooks.adelaide.edu.au/m/mill/john_stuart/m645o/chapter1.html

17 See e.g. House of Commons Science and Technology Committee. (2005). *Inquiry into human reproductive technologies and the law*. Fifth Report of Session 2004–05 Volume I. London: The Stationary Office; Nuffield Council on Bioethics. (2012). *Novel techniques for the prevention of mitochondrial DNA disorders: An ethical review*. London: Nuffield Council on Bioethics; Barber, S., & Border, P. (2015). *Mitochondrial donation*. SN/SC/6833, House of Commons Library Research Briefing.Retrieved from https://researchbriefings.parliament.uk/ResearchBriefing/Summary/SN06833#fullreport

18 See s61 Human Fertilisation and Embryology Act 2008 providing the Secretary of State with the power to make an order or regulations under the Act and The Human Fertilisation and Embryology (Mitochondrial Donation) Regulations 2015. See also HC Deb 3 February 2015, vol 592, cols 160–169 and HL Deb 24 February 2015, vol 759, cols 1569–1572

19 Nuffield Council on Bioethics. (2012). *Novel techniques for the prevention of mitochondrial DNA disorders: An ethical review*. London: Nuffield Council on Bioethics.

20 Nedelsky, J. (1989). Reconceiving autonomy: Sources, thoughts and possibilities. *Yale Journal of Law and Feminism, 1*(1), 7–36.

21 See for example, work by Nedelsky, *op. cit*. note 20; O’Neill, O. (2002). *Autonomy and trust in bioethics*. Cambridge: Cambridge University Press; Jackson, *op. cit*. note 12.

22 Jackson, op. cit. note 12.

23 Ibid., p. 4.

24 Ibid., p. 7.

25 Dworkin, R. (1993). *Life’s dominion: An* *argument about abortion and euthanasia*. London: Harper Collins.

26 Robertson, J. A. (1994). *Children of choice: Freedom and the new reproductive technologies*. Princeton, NJ: Princeton University Press.

27 Ibid., p. 22.

28 Ibid., p. 30.

29 Ibid.

30 See *Re T (Adult Refusal of Medical Treatment)* [1992] 3 WLR 782 and *Re MB (Medical Treatment)* [1997] 2 FLR 426, although attention should be drawn to case law where patient autonomy has been violated in cases of forced caesarean section, for example in *St. George’s Healthcare NHS Trust v *S, *R v Collins and Others Ex parte *S (1998), where the Court of Appeal held that a hospital acted unlawfully in forcing a pregnant woman to give birth by caesarean section against her wishes, decided following the earlier case of *Re MB*.

31 Robertson, *op. cit.* note 26, p. 264.

32 Raz, *op. cit.* note 11.

33 Jackson, *op. cit.* note 12*, *p. 8.

34 Note that in 2017, it was reported by Fertility Network UK that seven CCGs had decommissioned IVF services altogether, with only 12% offering the three treatment cycles recommended by NICE. See Fertility Fairness. (2017, October 30). Number of CCGs offering 3 IVF cycles has halved since 2013. Retrieved from https://www.fertilityfairness.co.uk/number-of-ccgs-offering-3-ivf-cycles-has-halved-since-2013/

35
*R (on the application of Burke) v General Medical Council & Ors* [2005] EWCA Civ 1003 [31].

36 Whether the state *should* or *ought* to provide universal access to fertility treatment is beyond the scope of this analysis.

37 On this point, see *R (on the Application of Mellor) v. Secretary of State for the Home Department *[2001] EWCA Civ 472, where the court held that, ‘it should be noticed at the outset that the obligation on the state is to respect family life: it does not allow persons to claim a right to *establish* family life, e.g. by marrying or having the opportunity to have children’ (emphasis my own). See also *SH and Others v Austria* [2011] ECHR 57813/00, where the European Court of Human Rights held that although the object of the Article 8 right to family life is essentially that of protecting the individual against arbitrary interference by public authorities, in addition to this primarily negative undertaking, there may be positive obligations inherent in an effective respect for private and family life. However, the Court also noted that Article 8(2) provides states with a wide margin of appreciation due to the sensitive nature of the issue of reproduction. [87].

38 Walsh, F. (2018, June 5). First UK womb transplant by ‘end of 2018’. *BBC*. Retrieved from https://www.bbc.co.uk/news/health-44360786

39 Given the limited scope of this paper, the purpose of this section is to briefly map out some of the main concerns with live donation in the context of the harm/benefit assessment. For a more detailed analysis of the relevant issues, see Williams, N. (2016). Should deceased donation be morally preferred in uterine transplantation trials? *Bioethics*, 30(6), 415–424 and Kisu, I., Mihara, M., Banno, K., Umene, K., Araki, J., Hara, H. … Aoki, D. (2013). Risk for donors in uterus transplantation. *Reproductive Sciences*, *20*(12), 1406–1415, p. 1409.

40 Williams, N., *op. cit.* note 39, p. 418

41 Brännström, M., Johannesson, L., Dahm‐Kähler, P., Enskog, A., Mölne, J., Kvarnström, N., Diaz‐Garcia, C., Hanafy, A., Lundmark, C., … Olausson, M. (2014). First clinical uterus transplantation trial: A six‐month report. *Fertility and Sterility*, *101*(5), 1228–1236, p. 1233.

42 Kisu, I., Kato, Y., Obara, H., Matsubara, K., Matoba, Y., Banno, K., & Aoki D1. (2018). Emerging problems in uterus transplantation. *International Journal of* *Obstetrics and Gynecology*, 1–5. https://doi.org/10.1111/1471-0528.15230

43 Wei, L., Xue, T., Tao, K. S., Zhang, G., Zhao, G. Y., Yu, S. Q., Cheng, L., … Chen, B.L. (2017). Modified human uterus transplantation using ovarian veins for venous drainage: The first report of surgically successful robotic‐assisted uterus procurement and follow‐up for 12 months. *Fertility and Sterility, 108*(2), 346–356.e1.

44 Kisu, I., et al., *op. cit*. note 42, p. 3.

45 Ibid., p. 3

46 Kisu, I., Mihara, M., Banno, K., Umene, K., Araki, J., Hara, H., Suganuma, N., & Aoki, D. (2013). Risk for donors in uterus transplantation. *Reproductive Sciences, 20*(12), 1406–1415, p. 1409.

47 Ibid.

48 Lavoué, V., Vigneau, C., Duros, S., Boudjema, K., Levêque, J., Piver, P., Aubard, Y., & Gauthier, T. (2017). Which donor for uterus transplants: Brain‐dead donor or living donor? A systematic review. *Transplantation* *, 101*(2), 267–273.

49 See the provisions of the Human Tissue Act 2004; the Human Tissue (Scotland) Act 2006; Sections 9–14 of the Human Tissue Act (Persons who Lack Capacity to Consent and Transplants) Regulations 2006 and the Human Tissue Authority. (2017). Donation of solid organs and tissues for transplantation code of practice. Retrieved from https://www.hta.gov.uk/sites/default/files/files/HTA%20Code%20F.pdf

50 Human Tissue Authority. (2017). *Guidance for transplant teams and independent assessors*. London: Human Tissue Authority. Retrieved from https://www.hta.gov.uk/sites/default/files/Guidance%20for%20Transplant%20Teams%20and%20Independent%20Assessors.pdf

51 Op. cit. note 41.

52 Daar, J., & Klipstein, S. (2016). Refocusing the ethical choices in womb transplant. *Journal of Law and the Biosciences, 3*(2), 383–388, p. 385.

53 Ibid., p. 387.

54 Ibid., p. 388.

55 See Wiles, K. S., Tillett, A. L., & Harding K. R. (2016). Solid organ transplantation in pregnancy. *The Obstetrician & Gynaecologist*, *18*, 189–197 for further data on maternal and fetal pregnancy risks and outcomes in solid organ transplant recipients.

56 McKay, D. B., & Josephson, M. A. (2006). Pregnancy in recipients of solid organs – Effects on mother and child. *The New England Journal of Medicine, 354*(12), 1281–1293, p. 1288.

57 Ibid. See also Deshpande, N. A., Coscia, L. A., Gomez‐Lobo, V., Moritz, M. J., & Armenti, V. T. (2013). Pregnancy after solid organ transplantation: A guide for obstetric management. *Reviews in Obstetrics & Gynecology,* *6*(3‐4), 116–125 and Wiles, K. S., MacDorman, M.F., Rasmussen, S. A., Qin, C., & Lackritz, E.M. *op. cit.* note 55.

58 See for example McIntire, D. D. (1999). Birth weight in relation to morbidity and mortality among newborn infants. *The New England Journal of Medicine, 340*, 1234–1238 and Callaghan W. M. et al. (2006). The contribution of preterm birth to infant mortality rates in the United States. *Pediatrics, 118*(4), 1566–1573.

59 McKay, *op. cit*. note 56.

60 See Brännström, M., et al., *op. cit.* note 4, and Brännström, M., Bokström, H., Dahm‐Kähler, P., Diaz‐Garcia, C., Ekberg, J., Enskog, A., Hagberg, H., Johannesson, L., … Rodriguez‐Wallberg, K. (2016). One uterus bridging three generations: First live birth after mother‐to‐daughter uterus transplantation. *Fertility and Sterility, 106*(2), 261–266.

61 Brännström, M., et al., *op. cit.* note 4, p. 613.

62 Brännström, M., et al., *op. cit.* note 51, p. 264.

63 Gustafsson Kubista, M. (2017, September 15). *Eight children born after uterus transplants*. University of Gothenburg Research. Retrieved from https://www.gu.se/english/research/news-detail//eight-children-born-after-uterus-transplants.cid1516702

64 Brännström, M., et al., *op. cit.* note 41, p. 1229.

65 Brännström, M., et al., *op. cit.* note 51, p. 264.

66 Ibid.

67 Where that patient has the requisite capacity – see *Re T*., *op. cit.* note 30.

68 See *Paton v British Pregnancy Advisory Service Trustees* [1979] QB 276 at [279].

69 The extent or nature of which I do not intend to explore here.

70 Golombok, S., Readings, J., Blake, L., Casey, P., Marks, A, & Jadva V. (2011). Families created through surrogacy: Mother–child relationships and children’s psychological adjustment at age 7. *Developmental Psychology, 47*(6), 1579–1588.

71 Ibid.

72 See Saso, S. et al., *op. cit. *note 6, p. 506.

73 Kumar Deka, P., & Sarma, S. (2010). Psychological aspects of infertility. *British Journal of Medical Practitioners*, *3*(3), a336.

74 Johannesson, L., & Järvholm, S. (2016). Uterus transplantation: Current progress and future prospects. *International Journal of Women’s Health, 8*, 43–51, p. 48

75 Järvholm, S., Johannesson, L., Clarke, A., & Brännström, M. (2015). Uterus transplantation trial: Psychological evaluation of recipients and partners during the post‐transplantation year. *Fertility and Sterility*, *104*(4), 1010–15, p. 1014.

76 Ibid., p. 1013.

77 Saso, S. et al., *op. cit.* note 2.

78 Ibid., p. 30.

79 Ibid.

80 Indeed, although trials currently require women to have the required number of embryos conceived of their own and not of donor ova, equating to both a genetic and a gestational experience, this does not necessarily preclude the use of donor eggs in treatment moving forward.

81 Note that the sample contained only 40 interviewees who volunteered to sign up as part of the clinical trial.

82 Saso, S. et al., *op. cit.* note 2, p. 32.

83 I thank Reviewer 1 for this example.

84 See Guidance Note 7 of the HFEA Code of Practice 9th edn. (2018). Retrieved from https://www.hfea.gov.uk/media/2565/hfea-draft-code-of-practice-9th-edition-consultation-version.pdf and the HFEA one at a time campaign aiming to reduce the multiple birth rate in IVF: Human Fertilisation and Embryology Authority. (n.d). *Our campaign to reduce multiple births*. Retrieved from https://www.hfea.gov.uk/about-us/our-campaign-to-reduce-multiple-births/

85 Although note that the literature supports the idea of changing embryo transfer policy towards one embryo transfer without any remarkable decrease in the success rate, e.g., Martikainen, H., Tiitinen, A., Tomás, .C., Tapanainen, J., Orava, M., Tuomivaara, L., … Vilska, S. (2001). One versus two embryo transfer after IVF and ICSI: A randomized study. *Human Reproduction, 16*(9) 1900–1903.

86 See for example guidance issued by the U.S. Department of Health and Human Services, National Institutes of Health (n.d). *What are some of the common complications of pregnancy?* Retrieved from https://www.nichd.nih.gov/health/topics/pregnancy/conditioninfo/Pages/complications.aspx

87 Lotz, M. (2016). Commentary on Nicola Williams and Stephen Wilkinson: ‘Should uterus transplants be publicly funded?’ *Journal of Medical Ethics, 42*(9), 570.

88 See for example, Lublin, N. (1998). *Pandora’s box: Feminism confronts reproductive technology*. New York, NY: Rowman & Littlefield; O’Neill, O. (2001). *Autonomy and trust in bioethics*. Cambridge: Cambridge University Press; Robertson, *op. cit.* note 26.

89 See for example, Corea, G., Duelli Klein, R., Hanmer, J., Holmes, H.B., Hoskins, B., Kishwar, M. … Steinbacher, R. (Eds). (1987) *Man made women: How new reproductive technologies affect women*. Bloomington: Indiana University Press; Purdy, L. (1996). *Reproducing persons: Issues in feminist bioethics*. New York, NY: Cornell University Press.

90 Lotz, *op. cit.* note 87, p. 570.

91 Ibid.

92 Russo, N.F. (1976). The motherhood mandate. *Journal of Social Issues, 32*(3), 143–153.

93 See Petropanagos, *op. cit.* note 3.

94 Saso, *op. cit.* note 2.

95 Ibid, Appendix B.

96 Human Fertilisation and Embryology Authority. (2016). *Fertility treatment 2014: Trends and figures*. Retrieved from https://www.hfea.gov.uk/media/1783/fertility-treatment-2014-trends-and-figures.pdf, 49

97 Jackson, *op. cit.* note 12, p. 3.

